# Experimental Study on the Axial Tensile Properties of FRP Grid-Reinforced ECC Composites

**DOI:** 10.3390/ma14143936

**Published:** 2021-07-14

**Authors:** Langni Deng, Lizhen Lei, Shijin Lai, Ling Liao, Zheng Zhou

**Affiliations:** School of Civil Engineering and Architecture, Guangxi University of Science and Technology, Liuzhou 545006, China; langni666@126.com (L.D.); leilizhen666@163.com (L.L.); laishijin666@163.com (S.L.); zz747377020@163.com (Z.Z.)

**Keywords:** textile-reinforced mortar, composite materials, axial tensile properties, stress–strain intrinsic relationship

## Abstract

The axial tensile properties of FRP mesh-reinforced ECC composites (TRE) were investigated experimentally under the consideration of four influencing factors: grid type, number of reinforcement layers, ECC matrix thickness, and sticky sand treatment on the grid surface. The test results showed that the axial stiffness and tensile strength of the composite were significantly increased, and the tensile properties were significantly improved under the effect of FRP grid reinforcement. Increasing the thickness of the ECC matrix can obviously improve the crack resistance of composites. The ultimate tensile strength of FRP lattice-reinforced ECC composites increased significantly with the increase in the number of lattice layers, but had no significant effect on the crack resistance. The tensile properties of CFRP grid-reinforced ECC composites were slightly better compared to BFRP grid-reinforced ECC composites. The crack resistance and ultimate tensile strength of the composites were slightly improved by impregnating the surface of the FRP grid with adhesive-bonded sand treatment. Based on the experimental data, the tensile stress–strain constitutive model of FRP grid-reinforced ECC composites is established. The calculation results show that the theoretical values of the model agree well with the experimental values. Therefore, it can be used to reflect the stress–strain change state of FRP lattice-reinforced ECC composites during axial tension.

## 1. Introduction

Fiber reinforced polymer (FRP) is favored in the field of concrete structure reinforcement engineering for its light weight and high strength, easy construction, corrosion resistance and durability. FRP is generally reinforced with organic resin adhesives. However, under the influence of long-term ultraviolet rays, humidity, high temperature or fire, the organic resin adhesive is prone to aging, which leads to the degradation of the bonding performance between FRP materials and concrete, and seriously reduces the reinforcement effect of FRP materials [1,2,3]. In order to make up for the defects of FRP material paste reinforcement method, in the 1980s, some scholars tried to replace organic resin adhesive with inorganic material and proposed textile reinforced mortar (TRM) [4]. TRM not only has good adhesion with concrete, but also has the advantages of high tensile strength and high temperature resistance, so it is applied to the research and testing of concrete structure reinforcement [3,4,5,6]. However, with the continuous research, research scholars found that TRM was a brittle material. Under loads, the crack control ability of TRM was low, with a crack control range of 0.1–0.4 mm and tensile fracture strain of about 0.5%, which was only 0.17–0.5 times of the fracture strain of the FRP grid, resulting in the fact that the high strength properties of the FRP grid were difficult to fully utilize [7,8].

Engineered cementitious composites (ECCs) are inorganic cement-based materials reinforced by a short fiber which is less than 2.5% of the total volume of the material [9]. Under bending or tensile load, ECCs present the development mode of multi-point cracking and fine cracks, and their ultimate strain can reach more than 3%, with good ductility and toughness [10,11]. ECCs have higher toughness and durability than ordinary mortar. If ECCs are used instead of ordinary mortar substrate in the TRM strengthening method, it is expected to overcome the shortcomings of poor crack resistance and the low ductility of traditional TRM, and further fully utilizes the high strength performance of the FRP grid. Therefore, some scholars combined the FRP grid with ECCs to form textile reinforced ECC (TRE). Xu et al. [12] studied the flexural properties of carbon textile reinforced ECCs, and found that sand adhesion on the surface of the carbon fiber significantly improved the cracking and ultimate flexural strength of the specimens. Wang et al. [13,14] studied the tensile mechanical properties of basalt grid-reinforced ECC composites and found that a basalt grid can significantly improve the axial stiffness and ultimate bearing capacity of ECCs, and the higher the number of layers, the greater the improvement. Jiang et al. [15] used basalt textile grid-reinforced ECC composites and found that the best tensile performance of the composites was achieved at an ECC matrix thickness of 3 mm; ECCs could improve the reinforcement of the BFRP grid better than ordinary mortar. Li et al. [16] investigated the tensile and flexural properties of two types of hybrid woven grid-reinforced ECC composites, a carbon–glass grid and basalt–glass grid, and found that the ultimate tensile and flexural strengths and ductility of the carbon–glass grid-reinforced specimens were superior compared to those of the basalt–glass grid-reinforced specimens.

At present, the tensile mechanical properties of CFRP grid-reinforced ECC composites are less studied in the study of tensile mechanical properties of FRP lattice-reinforced ECC composites. Compared with the BFRP grid, the CFRP grid has higher strength and deformability, so developing the mechanical properties of ECC composites reinforced by the CFRP grid is significant. Therefore, based on the existing studies, this paper mainly considers the effects of grid type (CFRP grid and BFRP grid), the number of grid reinforcement layers, the ECC matrix thickness and grid surface impregnated with adhesive sand treatment on the tensile mechanical properties of reinforced specimens, and explores the stress–strain development law of TRE under axial tensile loading.

## 2. Experiment Design

### 2.1. Specimen Design

**Figure 1:**
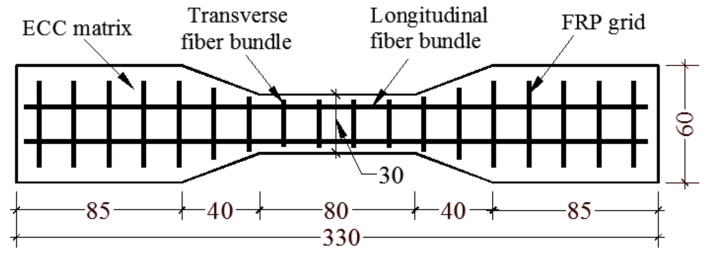
Schematic design of FRP grid-reinforced ECC uniaxial tensile specimen.

A total of 11 groups were designed for the test, with 3 specimens in each group. One group was the ECC specimen without grid reinforcement as the test control group, and the rest were the FRP grid-reinforced ECC composite specimen group. The specimen design parameters are shown in Table 1, which mainly consider the effects of different FRP grid types (CFRP grid and BFRP grid), the number of FRP grid reinforcement layers, the thickness of the ECC matrix and the surface of the mesh dipping adhesive sand treatment on the axial tensile properties of the composite specimen. The profile shape of the tensile specimen was designed to be dumbbell shaped. The main purpose was to prevent the specimen from slipping at the clamping end and damage due to stress concentration during loading [17,18]. The specimen profile dimensions were designed as shown in Figure 1. The dumbbell-type specimen consisted of a variable section area with a clamping section of 125 mm in length and a measuring section of 80 mm.

### 2.2. Test Raw Materials

The main material components of the ECC matrix were Class I fly ash, silicate cement P.O 42.5, quartz sand of 100~15 mesh, silica fume with an average particle size of 0.1~0.3 μm, a water reducing rate of 30%, a PCA-type polycarboxylic acid high efficiency water reducing agent, and PVA fiber with lengths of about 12 mm. The fly ash, silica fume, quartz sand and water reducing agents are produced by Qingyang Water Treatment Materials Effective Company in the city of Gongyi, Henan Province, China; PVA fiber is produced by Kuraray Fibers Ltd. in Tokyo, Japan, and cement is produced by Yufeng Cement Plant in Liuzhou City, Guangxi Province, China. The ECC coordination is shown in Table 2. The performance parameters of PVA fiber materials are shown in Table 3.

**Figure 2:**
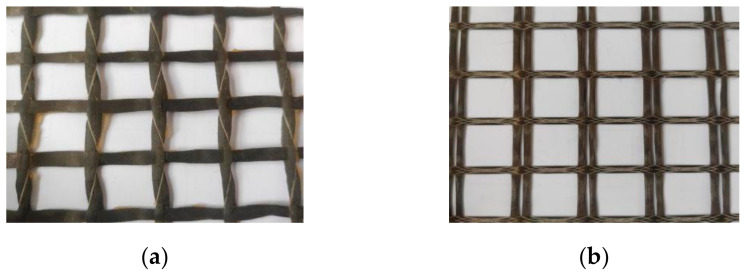
FRP grid sample: (**a**) CFRP grid. (**b**) CFRP grid.

The CFRP grid (Figure 2a) mainly used in the experiment is produced by Tianjin Kaben Technology Group Co., Ltd., Tianjin, China. The grid of BFRP (Figure 2b) is produced by Jiangsu Green Wood Valley Co., Ltd., Jiangsu, China. The geometric parameters of the two types of FRP meshes are shown in Table 4. With reference to the literature [19], the ETM105D microcomputer-controlled electronic universal testing machine (Wanchen Testing Machine Co. Ltd., Jinan, China) was used to carry out the tensile performance test of the FRP grid to obtain its basic mechanical parameters. The average ultimate tensile strengths of the CFRP grid and BFRP grid were measured to be 4255 Mpa and 597.1 Mpa, the average ultimate tensile strains were 1.75% and 2.28%, and the average modulus of elasticity were 243.1 Gpa and 26.18 Gpa, respectively.

### 2.3. Test Setup

**Figure 3:**
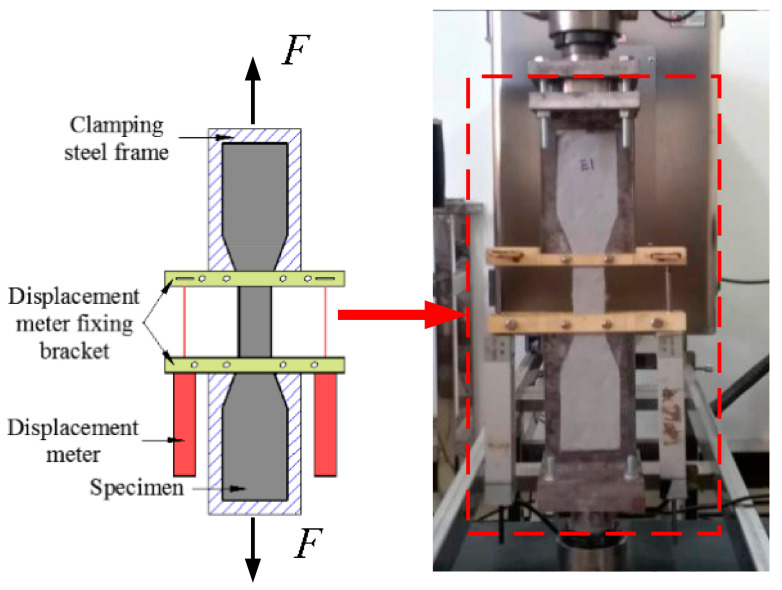
Schematic diagram of specimen loading device.

The test loading setup is shown in Figure 3. Two displacement transducers were fixed on both sides of the specimen measurement area to measure the axial tensile deformation of the specimen. The specimen measurement distance was 80 mm. The experiment was carried out by using an ETM105D microcomputer-controlled electronic universal testing machine for displacement control loading with a loading rate of 0.3 mm/min. Axial displacement meter data were automatically collected using JM3841's static signal analysis system (Wanchen Testing Machine Co. Ltd., Jinan, China) with a 50 Hz acquisition frequency.

## 3. Analysis of Experimental Results

### 3.1. Experimental Phenomenon

**Figure 4:**
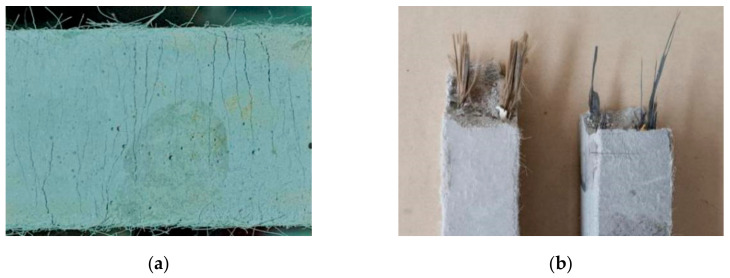
Specimen tensile damage pattern. (**a**) Multi-crack development characteristics of specimen surface, (**b**) FRP grid-reinforced ECC specimen pull-off.

For the ECC specimen group without FRP grid reinforcement (E0 group), when the external load reached the cracking load of the ECC specimen, transverse fine cracks perpendicular to the loading direction began to appear on the specimen surface [20]. With the continuous increase in external load, new fine cracks continuously sprouted on the surface of the specimen, showing the characteristics of multiple fine cracks (Figure 4a), and the original cracks gradually expanded along both sides of the specimen [19]. When the external load approached the ultimate load of the ECC specimen, the width of a fine crack on the surface of the specimen gradually increased and evolved into a main crack. When the external load reached the ultimate load of the ECC specimen, the specimen was eventually damaged by pulling out or pulling off the PVA fibers at the main crack section. For the FRP grid-reinforced ECC specimens, fine microcracks also appeared on the specimen surface when loaded to the cracking load of the specimens [15]. The crack development characteristics were basically the same as those of the ECC specimens without mesh reinforcement, but the number of cracks was higher than that of the latter. When loaded to the ultimate load of the specimen, the FRP grid embedded in the ECC matrix emitted a slight "pop" sound, followed by fracture at the main crack section, resulting in the destruction of the specimen [13]. However, it was observed that the BFRP grid-reinforced ECC specimens had a faster rate of main crack width expansion and a faster rate of damage compared to the CFRP grid-reinforced ECC specimens. The main reason is that the tensile strength of the BFRP grid is lower compared to the CFRP grid, which results in a lower tensile capacity of the specimen. The damage patterns of the specimens were PVA fiber pull-out or pull-off and longitudinal fiber bundle breakage of the FRP grid (see Figure 4b), but no significant relative slip occurred at the interface between the FRP grid and the ECC matrix [14].

### 3.2. Stress–Strain Relationship Curve

**Figure 5:**
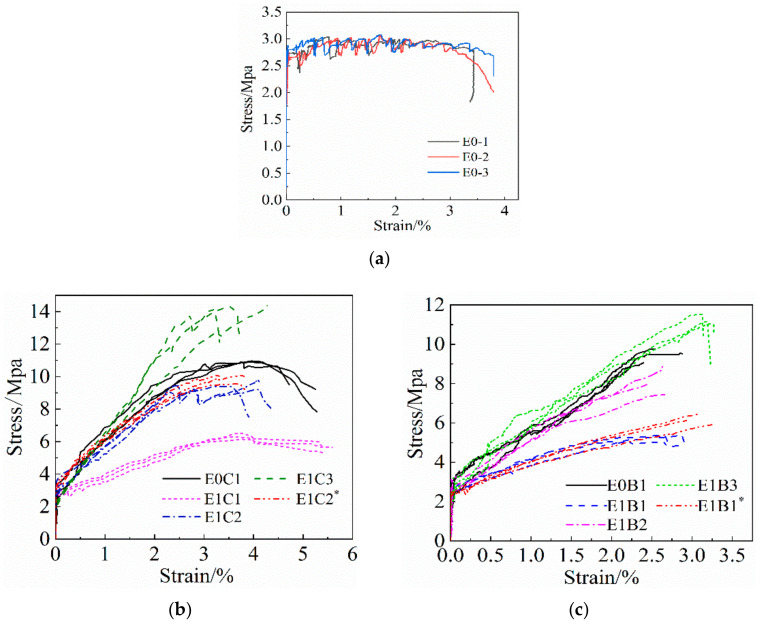
Specimen axial tensile stress–strain curve. (**a**) ECC specimen set, (**b**) CFRP grid-reinforced ECC specimen set, (**c**) BFRP grid-reinforced ECC specimens.

In Figure 5, the stress–strain curves of all tensile specimens are plotted. From the figure, it can be seen that the stress–strain curves of the specimens can be divided into three stages, i.e., the elastic stage, multi-crack developmental stage and the damage stage.

(1) Elastic stage. At the beginning of loading, the specimen did not show cracks, the stress and strain basically kept a linear growth relationship, and the specimen was in the elastic working stage. By comparison, it was found that the slope of the curves of unreinforced ECC specimens and FRP mesh-reinforced specimens differed very little during this phase. This indicates that before the cracking of the reinforced specimens, the FRP grid contributes less to the force, although it cooperates with the ECC.

(2) Multi-crack development stage. When the ECC matrix is cracked, the specimen enters the second working stage. For the ECC specimens cracked without grid reinforcement, the tensile strain continued to grow, but the tensile stress fluctuated up and down within a certain value of development, and the increase was not obvious. The slope of its stress–strain curve was almost close to horizontal, and the specimen entered the stable development stage of multi-point cracking, showing high ductility characteristics. The main reason was that after ECC cracking, the PVA fibers between the cracks and the cement matrix underwent relative slippage and did not break or pull out yet, and could still transfer part of the tensile stress. It played a toughening effect on the cracks and effectively inhibited the crack expansion, which made the specimens show a fine crack development pattern and strain strengthening characteristics. For the FRP mesh-reinforced ECC specimen group, when the ECC matrix cracked, the growth rate of the tensile strain of the reinforced specimen was significantly larger than the growth rate of stress, and the slope of the cut line of the curve gradually decreased. This indicates that the tensile stiffness of the reinforced specimens gradually declines. During this phase, the FRP grid shared most of the tensile stress of the PVA fibers, which inhibited the rate of PVA fiber pull-out or fracture from the cement matrix at the crack cross-section, making the number of cracks in the specimens increase significantly. The axial tensile stiffness of the specimens was enhanced, which effectively compensated the deficiency of the tensile strength of the ECC matrix. In addition, the slope of the curve for the FRP grid-reinforced ECC composite specimens within this phase was significantly larger than that for the unreinforced ECC specimens, indicating that the tensile stresses within this phase are mainly borne by the FRP grid and the contribution of the ECC matrix is smaller. Comparing Figure 5b,c, it was found that the slope of the curve is larger for the CFRP grid-reinforced ECC specimens within this stage compared to the BFRP grid-reinforced ECC specimens. This indicates that the tensile stiffness of the CFRP grid-reinforced ECC specimens is slightly better than that of the BFRP grid-reinforced ECC specimens.

(3) Damage stage. When the external load reached the ultimate tensile stress of the specimen, a main crack was produced on the surface of the specimen. The cement matrix at the cross-section of the main crack withdrew from the work, and the tensile stress was all borne by the FRP grid and PVA fiber. At this time, the tensile stress decreased, but the strain continued to increase, and the specimen broke. As found in Figure 5b,c, compared with the BFRP grid-reinforced ECC specimens, the slope of the curve decreases more slowly after the ultimate stress is reached in the CFRP grid-reinforced ECC specimens, indicating that the specimens are damaged more slowly. The main reason is that the tensile strength of the CFRP grid is higher than that of the BFRP grid.

### 3.3. Analysis of Influencing Factors

**Figure 6:**
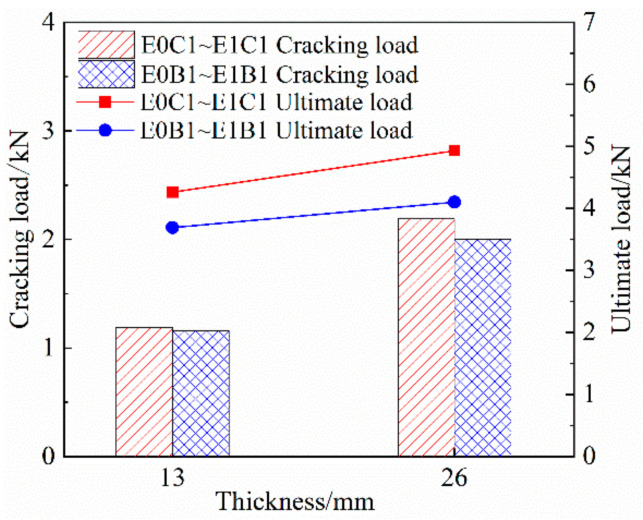
Effect of ECC substrate thickness on tensile properties of specimens.

**Figure 7:**
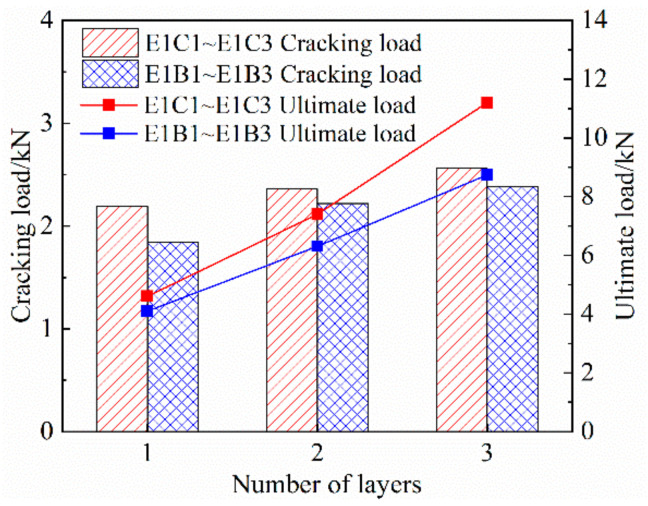
Effect of the number of layers of FRP grid reinforcement on the tensile properties of specimens.

**Figure 8:**
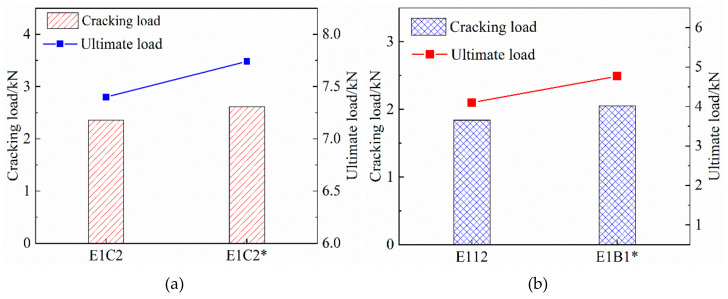
Effect of FRP grid surface impregnated with adhesive sand treatment on the tensile properties of specimens. (**a**) CFRP grid surface sticky sand treatment, (**b**) BFRP grid surface sticky sand treatment.

The critical values for the measured tensile results of the specimens were determined by the test phenomena and stress–strain curves and plotted in Table 5. The cracking stress is the tensile stress from the elastic stage to the inflection point of the multi-crack development stage to the damage stage. The ultimate stress is the tensile stress from the multi-crack development stage to the inflection point of the damage stage. Based on Table 5, the comparative axial extension properties of FRP grid-reinforced ECC specimens were plotted under each influencing factor, as shown in Figure 6, Figure 7 and Figure 8.

#### 3.3.1. Effect of ECC Substrate Thickness on Tensile Properties of Specimens

As shown in Figure 6 and Table 5, the cracking load and ultimate load of the composite specimens both tended to increase with the increase in the ECC matrix thickness. Among them, the average values of the cracking load growth of specimens E1C1 and E1B1 were 84.03% and 58.62%, and the average values of the ultimate load growth were 8.21% and 11.11%, respectively. This shows that the ECC matrix thickness has a significant effect on the cracking resistance of the specimens, but the effect on the ultimate tensile capacity of the specimens is small. The reason for analysis is that before the specimen cracks, the tensile stress is mainly borne by the ECC matrix and increasing the thickness of the ECC matrix is beneficial to enhance the tensile stiffness of the specimen and improve the crack resistance. After the specimen cracking, the tensile stress is mainly borne by the FRP grid, and the PVA fiber length is less than the thickness of the matrix, which leads to the bridge linkage being weakened, which is not conducive to improving the tensile stiffness of the specimen, and then the performance of increasing the thickness of the ECC matrix on the ultimate tensile capacity of the specimen improvement effect is general. In addition, the cracking stresses of specimens E1C1 and E1B1 decreased by 7.00% and 21.14%, and the ultimate stresses decreased by 45.83% and 44.5%, respectively, as the thickness of the ECC matrix increased. This is because the increase in ultimate load is smaller than the increase in the cross-sectional area of the specimens, so the converted strength decreases.

#### 3.3.2. Effect of the Number of Grid Layers on the Tensile Properties of Specimens

As shown in Figure 7 and Table 5, the number of FRP grid reinforcement layers maintained a positive correlation with the cracking load and ultimate load of the reinforced specimens. Compared with specimen E1C1, specimens E1C2 and E1C3 cracking load growth mean values are 7.76% and 16.89%, respectively, and ultimate load growth mean values are 49.76% and 125.28%, respectively. Compared with specimen E1B1, the average values of the cracking load growth of specimens E1B2 and E1B3 were 10.51% and 19%, respectively, and the average values of ultimate load growth were 53.99% and 113.12, respectively. This shows that the number of FRP grid reinforcement layers has a small effect on the cracking load of the specimens, but has a significant effect on the ultimate load. The reason for analysis is that the contribution of the FRP grid is small within the elastic stage, and increasing the number of layers of FRP grid reinforcement has a general effect on the improvement of the crack resistance of the specimen. Additionally, the FRP mesh plays a strengthening role and becomes the main stressor within the multi-crack development stage. Increasing the number of layers can significantly improve the overall tensile stiffness of the specimen and enhance the ultimate tensile capacity of the specimen. In addition, comparison with Figure 6 further shows that the cracking load of FRP grid-reinforced ECC composites is mainly determined by the tensile strength of the ECC matrix, while the ultimate load is mainly determined by the number of FRP grid-reinforced layers.

#### 3.3.3. Effect of Grid Type on Tensile Properties of Specimens

As shown in Figure 7 and Table 5, the cracking loads of CFRP grid-reinforced specimens were 9.5%, 6.3% and 7.5% higher than those of BFRP grid-reinforced specimens at all levels of reinforcement layers, and the ultimate loads were 20.24%, 17.27% and 27.23% higher, respectively. It shows that the CFRP mesh has a better reinforcement effect on ECCs with the same ECC matrix thickness and number of reinforcement layers. On the one hand, compared with the BFRP grid, the mechanical properties of the CFRP grid are better, so its reinforcement effect on the crack resistance and ultimate tensile capacity of the specimen is better with the same number of reinforcement layers. On the other hand, when the BFRP grid is buried in the ECC matrix, the cement colloid will penetrate the interior of the BFRP grid, causing the fiber bundles to solidify and increasing the brittleness of the fiber bundles, which is not conducive to utilizing the performance of the BFRP grid, resulting in slightly poor tensile properties of the BFRP grid-reinforced specimens.

#### 3.3.4. Effect of the Tensile Properties of Specimens Treated with Gum-Impregnated Adhesive Sand on the Surface of the Grid

As shown in Figure 8 and Table 5, compared with specimen E1C2, the cracking load and ultimate load of specimen E1C2* were increased by 10.6% and 4.6%, respectively. Compared with specimen E1B1, the cracking load and ultimate load of specimen E1B1* were increased by 11.41% and 16.34%, respectively. It is shown that the surface of FRP mesh is treated with adhesive-impregnated sand to have an effect on the crack resistance and ultimate tensile capacity of the specimens. The reason for the analysis is that the surface of FRP mesh is treated with adhesive bonding sand, which improves the bonding performance with the interface of the ECC matrix, strengthens the synergistic force effect between the two, enhances the crack control ability, strengthens the overall tensile stiffness of the specimen, and thus, optimizes the tensile performance of the specimen [16].

## 4. Stress–Strain Intrinsic Structure Relationship Model

### 4.1. Model Construction

**Figure 9:**
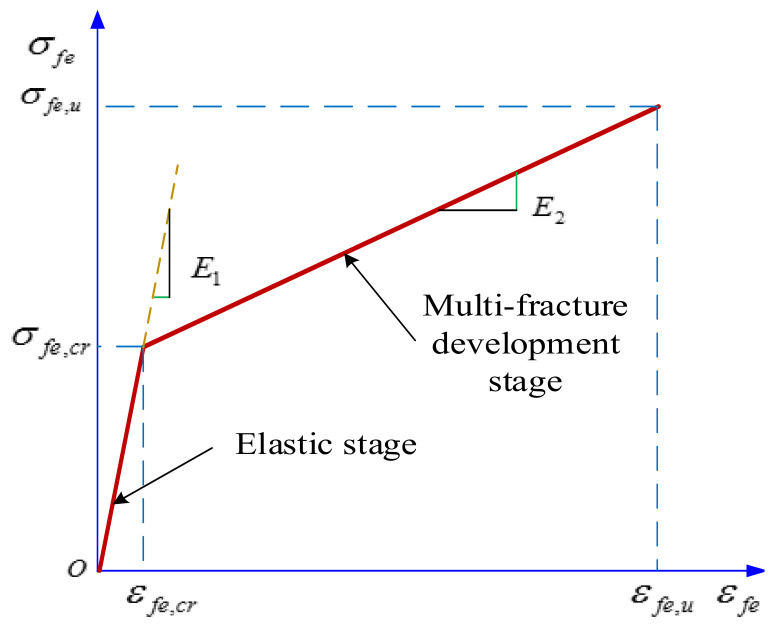
Uniaxial tensile stress–strain model for FRP grid-reinforced ECC composites.

From Figure 5, it can be seen that the force process of FRP grid-reinforced ECC specimens under axial tensile load is divided into three stages, which are: the elastic stage, multi-crack development stage and the damage stage. Since the curve characteristics of the damage phase are more discrete after the reinforced specimen reaches the ultimate tensile strength, this article mainly analyzes the stress–strain intrinsic relationship between the elastic phase and the strain-reinforced phase, and proposes a bilinear intrinsic relationship model, as shown in Figure 9. The test results showed that the stress–strain curve trend and characteristic point changes of FRP grid surface dipping-treated specimens were not obvious, and the parameter design was lower, which was not conducive to establishing parametric analysis. Therefore, this dual calculation model fails to fully consider the effect of the FRP grid surface impregnated with adhesive sand treatment on the tensile strength of the specimens. It is mainly applicable to reflect the tensile stress process of FRP grid-reinforced ECC specimens without the FRP grid surface impregnated with adhesive sand treatment. During the elastic phase, the specimen is not cracked yet, and the FRP grid is stressed in cooperation with the ECC matrix, the stress and strain maintain a linear growth relationship, and the slope of the curve mainly depends on the composite elastic modulus of the FRP grid and the ECC matrix. Within the multi-crack development stage, the stress and strain grow approximately linearly, but the growth rate of strain was significantly larger than the growth rate of stress. Additionally, the stress was mainly borne by the FRP grid, and the contribution of ECC matrix was smaller, so the slope of the curve was determined by the elastic modulus of the FRP grid. Therefore, the stress–strain intrinsic relationship model for FRP grid-reinforced ECC composites under axial tensile loading is as follows:

(1) $$\sigma_{fe}=\left\{\begin{array}{l} E_{1}\varepsilon_{fe},\varepsilon_{fe}\leq \varepsilon_{fe,cr}\\ E_{2}(\varepsilon_{fe}-\varepsilon_{fe,cr})+\sigma_{fe,cr},\varepsilon_{fe,cr}<\varepsilon_{fe}\leq \varepsilon_{fe,u} \end{array}\right.$$

In Equation (1), $\sigma_{fe}$ represents the stress of the composite material. $\sigma_{fe,cr}$ and $\sigma_{fe,u}$ are the cracking stress and ultimate stress of composite materials, respectively. $\varepsilon_{fe}$ is the strain of reinforced specimen. $\varepsilon_{fe,cr}$ and $\varepsilon_{fe,u}$ are the cracking strain and ultimate strain of composite materials, respectively. $E_{1}$ and $E_{2}$ are the curve slopes of composite materials in elastic stage and multi-crack development stage, respectively. Therefore, to calculate the tensile stress of FRP grid-reinforced ECC composites, the slope of the stress–strain curve, the cracking strain as well as the ultimate strain must be determined first.

### 4.2. Analysis of Model Parameters

**Figure 10:**
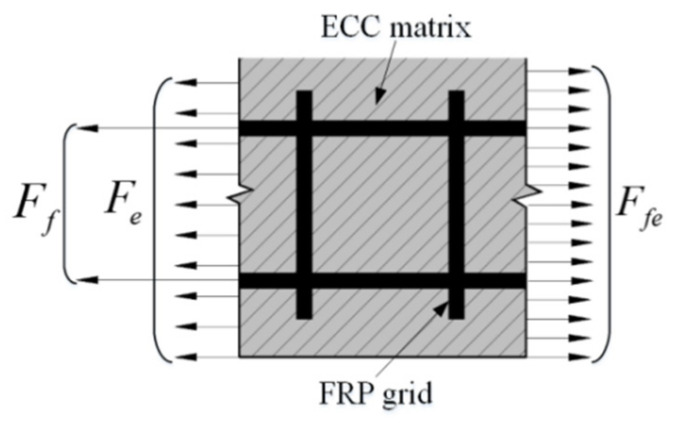
Cross-sectional force analysis of FRP grid-reinforced ECC composites.

The interface force analysis of FRP lattice-reinforced ECC composites is shown in Figure 10. When the composite material is in the elastic working phase, the FRP grid is stressed in cooperation with the ECC matrix, then according to the equilibrium condition, it is obtained that:

(2) $$F_{e}+F_{f}=F_{fe}$$

In Equation (2), $F_{e}$, $F_{f}$ and $F_{fe}$, respectively, represent the loads borne by the ECC matrix, FRP grid and composite material. According to the principle of material mechanics:

(3) $$\left\{\begin{array}{l} F_{e}=\sigma_{e}A_{e}\\ F_{f}=\sigma_{f}A_{f}\\ F_{fe}=\sigma_{fe}A_{fe} \end{array}\right.$$

In Equation (3), $\sigma_{e}$, $\sigma_{f}$, and $\sigma_{fe}$, respectively, represent the stresses in the ECC matrix, FRP grid, and composite material. $A_{e}$, $A_{f}$ and $A_{fe}$, respectively, represent the cross-sectional areas of ECC matrix, FRP grid and composite material. Combined with Equations (2) and (3):

(4) $$\sigma_{fe}=\sigma_{e}\rho_{e}+\sigma_{f}\rho_{f}$$

In Equation (3), $\rho_{e}=A_{e}/A_{fe}$, $\rho_{f}=A_{f}/A_{fe}$. In the elastic stage, the FRP mesh and ECC are stressed cooperatively, and they have good adhesion without relative slip. Therefore, it can be assumed that the reinforced specimen strain is equal to the FRP grid strain and the ECC matrix strain, and the cracking strain is equal to the ECC matrix cracking strain. That is, $\varepsilon_{fe}=\varepsilon_{f}=\varepsilon_{e}$, $\varepsilon_{fe,cr}=\varepsilon_{e,cr}$. In addition, the slope of the curve in this phase depends mainly on the composite elastic modulus of the FRP grid and the ECC matrix. Then, Equations (5) and (6) are obtained.

(5) $$E_{1}=\frac{d\sigma_{fe}}{d\varepsilon_{fe}}=\rho_{e}\frac{d\sigma_{e}}{d\varepsilon_{e}}+\rho_{f}\frac{d\sigma_{f}}{d\varepsilon_{f}}$$

(6) $$E_{1}=E_{fe}=E_{e}\rho_{e}+E_{f}\rho_{f}$$

In Equation (6), $E_{e}$, $E_{f}$ and $E_{fe}$, respectively, represent the elastic modulus of the ECC matrix, FRP grid and reinforced specimen.

**Figure 11:**
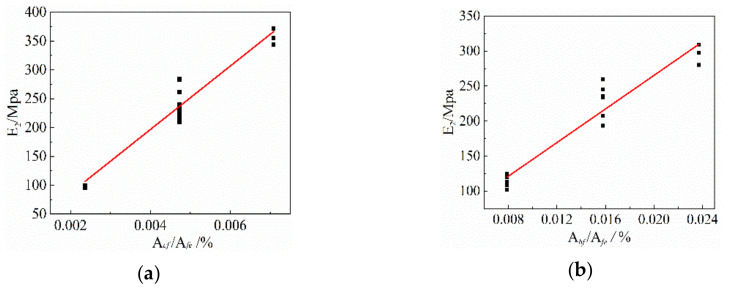
The relationship between the modulus of elasticity of FRP gird. (**a**) CFRP grid-reinforced ECC, (**b**) BFRP grid-reinforced ECC.

Since the stress of the composite material is mainly borne by the FRP grid and the ECC matrix has little contribution, the slope of the curve is mainly determined by the elastic modulus of the FRP grid. As shown in Figure 11, the regression analysis of the experimental data shows that the slope of the stress–strain curve $E_{2}$ within the strain-reinforced phase is related to the elastic modulus $E_{cf}$ of the CFRP mesh and the elastic modulus $E_{bf}$ of the BFRP mesh by the following regression equations.

(7) $$E_{2}=0.226E_{cf}\rho_{cf}-23$$

(8) $$E_{2}=0.458E_{bf}\rho_{bf}+25$$

In Equations (7) and (8), $\rho_{cf}=A_{cf}/A_{fe}$, $\rho_{bf}=A_{bf}/A_{fe}$. The experimental results show that the failure mode of the FRP grid-reinforced ECC material is the FRP grid fracture and the ultimate tensile strain $\varepsilon_{fe,u}$ is closer to the ultimate tensile strain $\varepsilon_{fu}$ of the FRP grid. Therefore, it can be set as:

(9) $$\varepsilon_{fe,u}=\beta_{i}\varepsilon_{fu}$$

In the Equation (9), $\beta_{i}$ indicates the correction factor for the ultimate strain of the composite specimen. Regression analysis yields a correction factor $\beta_{cf}$ = 1.82 for CFRP grid-reinforced ECC specimens, and a correction factor $\beta_{bf}$ = 1.226 for BFRP grid-reinforced ECC specimens.

According to Equation (3), the calculation models of the cracking load $F_{cr}$ and ultimate load $F_{u}$ of the FRP grid-reinforced ECC composite are as follows:

(10) $$\left\{\begin{array}{l} F_{cr}=\sigma_{fe,cr}A_{fe}\\ F_{u}=\sigma_{fe,u}A_{fe} \end{array}\right.$$

### 4.3. Model Validation

**Figure 12:**
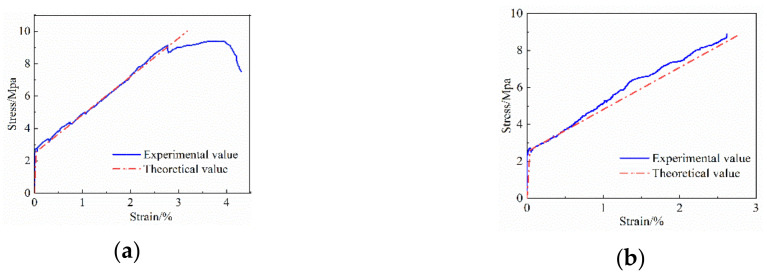
Comparison of stress–strain test values and theoretical values of the FRP grid-reinforced ECC composite specimens. (**a**) Specimen E1C2-2, (**b**) Specimen E1B2-3.

To verify the correctness of the stress–strain intrinsic structure relationship model of FRP grid-reinforced ECC composites established in this paper, the intrinsic structure relationship curves obtained from the tests of specimens E1C2-2 and E1B2-3 were selected for comparison with those calculated by Equation (1). As shown in Figure 12, the intrinsic structure relationship curve proposed in this paper is in good agreement with the experimental stress–strain curve trend. The test values of the cracking load and ultimate load of each specimen compared with the theoretical calculated values are shown in Table 6. The mean ratios of the cracking load and ultimate load test values to the theoretical values of FRP grid-reinforced ECC specimens were found to be in the range of 0.98~1.03, with the variance being less than 0.02 and the coefficient of variation less than 0.1. This indicates that the theoretical values of the FRP grid-reinforced ECC composites established in this paper are in good agreement with the measured values. Therefore, the FRP grid-reinforced ECC composites axially subjected to the tensile intrinsic model established in this paper can be used to reflect the stress–strain change state of FRP grid-reinforced ECC composites during axial tension.

## 5. Conclusions

In this paper, uniaxial tensile tests were conducted on FRP mesh-reinforced ECC composites to investigate the effects of the ECC matrix thickness, number of FRP mesh layers, different FRP mesh types (CFRP mesh and BFRP mesh), and the FRP mesh surface impregnated with adhesive sand treatment on the tensile properties of the specimens, and to establish the corresponding axial tensile stress–strain intrinsic structure relationship model. The conclusions are as follows:

(1) Before the specimen cracks, the FRP grid has little effect on enhancing the tensile properties of the specimen. After the specimen cracks, the FRP grid plays a strengthening role, which improves the strain strengthening performance of the ECC matrix, enhances the axial tensile stiffness and ultimate tensile capacity of the specimen, and effectively makes up for the lack of tensile strength of the ECC matrix.
(2) Increasing the thickness of the ECC matrix can significantly improve the crack resistance of the FRP grid-reinforced ECC specimens, but the ultimate tensile strength is generally improved.
(3) The ultimate tensile strength of FRP grid-reinforced ECC specimens can be significantly improved by increasing the number of grid reinforcement layers, but the improvement effect on crack resistance is general. In addition, the tensile properties of CFRP grid-reinforced ECC specimens are better than those of BFRP-reinforced ECC specimens under the same number of reinforcement layers.
(4) The crack resistance and ultimate tensile strength of FRP grid-reinforced ECC composite specimens are slightly improved by dipping the FRP grid surface with adhesive sand.
(5) Based on the experimental study, a constitutive model of axial tensile stress–strain of FRP grid-reinforced ECC composite specimens is proposed. The calculation results show that the theoretical values of the constitutive model are in good agreement with the experimental values.

## Figures and Tables

**Table 1:** FRP grid-reinforced ECC specimen design table.

Number	Specimen Number	Types of Grids	Number of Grid Layers	Thickness of Matrix/mm	Cross-Sectional Area/mm^2^
1	E0	/	/	13	390
2	E0C1	CFRP	1	13	390
3	E1C1	CFRP	1	26	780
4	E1C2	CFRP	2	26	780
5	E1C3	CFRP	3	26	780
6	E1C2 *	CFRP	2	26	780
7	E0B1	BFRP	1	13	390
8	E1B1	BFRP	1	26	780
9	E1B2	BFRP	2	26	780
10	E1B3	BFRP	3	26	780
11	E1B1 *	BFRP	1	26	780

Note: In Table 5, the specimen number mark * indicates that the FRP grid surface of the specimen was treated with epoxy resin adhesive for gluing sand.

**Table 2:** ECC matrix mix ratio (quality ratio).

Water	Cement	Fly Ash	Quartz Sand	Silica Fume	Water Reducer	PVA	Water Toglue Ratio	Sand to Glue Ratio
1.42	1	2.33	0.67	0.1	0.019	0.002	0.5	0.2

**Table 3:** Performance index of PVA fiber material.

Material Name	Diameter/μm	Length/mm	Density/g/cm^3^	Modulus of Elasticity/Gpa	Tensile Strength/Mpa	Elongation at Break/%
PVA	39	12	1.3	40	1530	7

**Table 4:** FRP grid geometry and basic mechanical property parameters index.

Grid Type	$S_{grid}(\mathrm{mm}\;\times \;\mathrm{mm})$	$A_{grid}/{\mathrm{mm}}^{2}$	$E_{grid}/\mathrm{Gpa}$	$f_{grid}/\mathrm{Mpa}$	$\varepsilon_{grid}/\%$
CFRP	20 × 20	0.921	243.1	4255	1.75
BFRP	25 × 25	3.082	26.18	597.1	2.28

Note: $S_{grid}$ is FRP grid size; $A_{grid}$ is FRP grid single bundle grid interface area; $E_{grid}$ is FRP grid modulus of elasticity; $f_{grid}$ is FRP grid ultimate tensile strength; $\varepsilon_{grid}$ is FRP grid ultimate tensile strain.

**Table 5:** Tensile test results of FRP grid-reinforced ECC specimens.

Specimen Number				Multi-Crack Development Stage		
	Cracking Load/kN	Cracking Stress/Mpa	Cracking Strain/10^−4^	Ultimate Load/kN	Ultimate Stress/Mpa	Extreme Strain/10^−2^
E0–1	1.07	2.74	4.20	1.18	3.03	3.16
E0–2	1.02	2.62	3.90	1.20	3.08	3.30
E0–3	1.10	2.82	4.05	1.18	3.03	2.85
Average	1.06	2.73	4.05	1.19	3.04	3.10
Coefficient of variation	0.04	0.04	0.04	0.01	0.01	0.07
E0C1–1	1.11	2.85	3.96	4.27	10.95	2.90
E0C1-2	1.32	3.38	4.20	4.25	10.90	2.70
E0C1-3	1.14	2.92	3.60	4.25	10.90	3.02
Average	1.19	3.05	3.92	4.26	10.91	2.87
Coefficient of variation	0.10	0.09	0.08	0.00	0.00	0.06
E1C1-1	2.09	2.68	3.95	4.76	6.10	3.15
E1C1-2	2.30	2.95	4.08	4.49	5.75	3.03
E1C1-3	2.19	2.81	3.65	4.59	5.88	3.24
Average	2.19	2.81	3.89	4.61	5.91	3.14
Coefficient of variation	0.05	0.05	0.06	0.03	0.03	0.03
E1C2-1	2.62	3.36	4.53	7.61	9.76	2.86
E1C2-2	2.33	2.99	3.86	7.25	9.29	3.33
E1C2-3	2.12	2.72	3.58	7.33	9.40	3.27
Average	2.36	3.02	3.99	7.40	9.48	3.15
Coefficient of variation	0.11	0.11	0.12	0.03	0.03	0.08
E1C3-1	2.54	3.26	3.43	11.15	14.29	3.65
E1C3-2	2.66	3.41	4.26	11.01	14.12	3.33
E1C3-3	2.48	3.18	4.07	11.20	14.36	3.64
Average	2.56	3.28	3.92	11.12	14.26	3.54
Coefficient of variation	0.04	0.04	0.11	0.01	0.01	0.05
E1C2*-1	2.52	3.23	3.85	7.89	10.12	3.11
E1C2*-2	2.49	3.19	3.76	7.88	10.10	3.34
E1C2*-3	2.83	3.63	4.00	7.44	9.54	3.07
Average	2.61	3.35	3.87	7.74	9.92	3.17
Coefficient of variation	0.07	0.07	0.03	0.03	0.3	0.06
E0B1-1	1.25	3.21	4.02	3.81	9.77	2.57
E0B1-2	0.96	2.46	3.72	3.72	9.54	2.91
E0B1-3	1.28	3.28	4.10	3.54	9.08	2.50
Average	1.16	2.98	3.95	3.69	9.46	2.66
Coefficient of variation	0.15	0.15	0.05	0.04	0.04	0.08
E1B1-1	1.93	2.47	3.70	4.18	5.36	2.60
E1B1-2	1.73	2.22	3.39	4.18	5.36	2.90
E1B1-3	1.85	2.37	3.62	3.93	5.04	2.20
Average	1.84	2.35	3.57	4.10	5.25	2.57
Coefficient of variation	0.10	0.13	0.16	0.14	0.18	0.35
E1B2-1	2.50	3.21	3.59	6.20	7.95	2.57
E1B2-2	1.94	2.49	3.75	6.93	8.88	2.73
E1B2-3	2.21	2.83	3.50	5.80	7.44	2.76
Average	2.22	2.84	3.61	6.31	8.09	2.69
Coefficient of variation	0.13	0.13	0.04	0.09	0.09	0.04
E1B3-1	2.32	2.97	3.99	8.95	11.47	2.99
E1B3-2	2.54	3.26	3.92	8.66	11.10	2.83
E1B3-3	2.28	2.92	4.20	8.61	11.04	2.80
Average	2.38	3.05	4.04	8.74	11.21	2.87
Coefficient of variation	0.06	0.06	0.04	0.02	0.02	0.04
E1B1*-1	2.03	2.60	3.97	4.78	6.13	2.81
E1B1*-2	1.95	2.50	3.78	4.90	6.28	3.00
E1B1*-3	2.15	2.76	3.86	4.64	5.95	2.91
Average	2.05	2.62	3.80	4.77	6.12	2.91
Coefficient of variation	0.05	0.05	0.03	0.03	0.03	0.03

Note: In Table 5, the specimen number mark * indicates that the FRP grid surface of the specimen was treated with epoxy resin adhesive for gluing sand.

**Table 6:** Comparison of test and theoretical values of FRP grid-reinforced ECC composite specimen load characteristic points.

Specimen Number	Cracking Load/kN			Ultimate Load/kN		
	Experimental Value $F_{cr,\exp}$	Theoretical Value $F_{cr,th}$	Fcr,expFcr,th	Experimental Value $F_{u,\exp}$	Theoretical Value $F_{u,th}$	Fu,expFu,th
E0C1-1	1.11	1.17	0.95	4.27	4.07	1.05
E0C1-2	1.32	1.24	1.06	4.25	4.14	1.03
E0C1-3	1.14	1.06	1.07	4.25	3.97	1.07
E1C1-1	2.09	2.24	0.93	4.76	4.86	0.98
E1C1-2	2.30	2.32	0.99	4.49	4.93	0.91
E1C1-3	2.19	2.08	1.06	4.59	4.69	0.98
E1C2-1	2.62	2.68	0.98	7.61	8.47	0.90
E1C2-2	2.33	2.28	1.02	7.25	8.08	0.90
E1C2-3	2.12	2.12	1.00	7.33	7.92	0.92
E1C3-1	2.54	2.18	1.16	11.15	11.17	1.00
E1C3-2	2.66	2.70	0.98	11.01	11.68	0.94
E1C3-3	2.48	2.58	0.96	11.20	11.56	0.97
E1C2*-1	2.52	2.28	1.11	7.89	8.08	0.98
E1C2*-2	2.49	2.22	1.12	7.88	8.03	0.98
E1C2*-3	2.83	2.36	1.20	7.44	8.16	0.91
Average μ	-	-	1.03	-	-	0.97
Variance σ	-	-	0.007	-	-	0.002
Coefficient of variation δ	-	-	0.078	-	-	0.06
E0B1-1	1.25	1.06	1.18	3.81	3.37	1.13
E0B1-2	0.96	0.98	0.98	3.72	3.29	1.13
E0B1-3	1.28	1.08	1.18	3.54	3.40	1.04
E1B1-1	1.93	2.13	0.91	4.18	4.70	0.89
E1B1-2	1.73	1.95	0.89	4.18	4.53	0.92
E1B1-3	1.85	2.08	0.89	3.93	4.66	0.84
E1B2-1	2.50	2.11	1.01	6.20	6.72	0.92
E1B2-2	1.94	2.07	0.99	6.93	6.68	1.04
E1B2-3	2.21	2.22	0.99	5.80	6.83	0.85
E1B3-1	2.32	2.13	1.09	8.95	8.78	1.02
E1B3-2	2.54	2.23	1.14	8.66	8.87	0.98
E1B3-3	2.28	2.08	1.11	8.61	8.73	0.99
E1B1*-1	2.03	1.94	1.04	4.78	4.52	1.06
E1B1*-2	1.95	1.79	1.08	4.90	4.37	1.12
E1B1*-3	2.15	1.86	1.15	4.64	4.44	1.05
Average μ	-	-	1.04	-	-	1.0
Variance σ	-	-	0.010	-	-	0.009
Coefficient of variation δ	-	-	0.097	-	-	0.097

Note: In Table 5, the specimen number mark * indicates that the FRP grid surface of the specimen was treated with epoxy resin adhesive for gluing sand.

## Data Availability

The data presented in this study are available on request from the corresponding author.

## References

[1] Lu Y.Y. (2018). Research progress of concrete structures strengthened with fiber reinforced composites and steel. J. Build. Struct..

[2] Ai S.X., Yin S.P., Xu S.L. (2015). Research progress and application of textile reinforced concrete. J. Civ. Eng..

[3] Triantafillou T.C., Papanicolaou C.G., Zissimopoulos P., Laourdekis T. (2006). Concrete Confinement with Textile-Reinforced Mortar Jackets. ACI Struct. J..

[4] Dai J.G., Munir S., Ding Z. (2014). Comparative Study of Different Cement-Based Inorganic Pastes towards the Development of FRIP Strengthening Technology. J. Compos. Constr..

[5] Raoof S.M., Koutas L.N., Bournas D.A. (2016). Bond between textile-reinforced mortar (TRM) and concrete substrates: Experimental investigation. Compos. Part B.

[6] Ding Z., Dai J.G., Muner S. (2014). Study on an Improved Phosphate Cement Binder for the Development of Fiber-Reinforced Inorganic Polymer Composites. Polymers.

[7] Soranakom C., Mobasher B. (2009). Geometrical and mechanical aspects of fabric bonding and pullout in cement composites. Mater. Struct..

[8] Carozzi F.G., Poggi C. (2015). Mechanical properties and debonding strength of Fabric Reinforced Cementitious Matrix (FRCM) systems for masonry strengthening. Compos. Part B Eng..

[9] Li V.C. (2002). Advances in ECC research. ACI Spec. Publ. Concr. Mater. Sci. Appl..

[10] Alyousif A., Lachemi M., Yildirim G., Aras G.H., Sahmaran M. (2016). Influence of Cyclic Frost Deterioration on Water Sorptivity of Microcracked Cementitious Composites. J. Mater. Civ. Eng..

[11] Hung C.C., Su Y.F., Su Y.M. (2018). Mechanical properties and self-healing evaluation of strain-hardening cementitious composites with high volumes of hybrid pozzolan materials. Compos. Part B Eng..

[12] Xu S.L., Li Q.H., Li H.D. (2007). Experimental study on flexural properties of ultra-high toughness cement-based composites reinforced by carbon fiber woven mesh. J. Civ. Eng..

[13] Zhu Z.F., Wang W.W. (2017). Mechanical properties test and constitutive relation model of basalt grid reinforced cement matrix composites under uniaxial tension. Acta Compos. Sin..

[14] Zheng Y.H., Wang W.W., Li J.F., Han G. (2016). Tensile constitutive model of composite grid-high ductility fiber cement base. Ind. Archit..

[15] Li B., Xiong H., Jiang J., Dou X. (2018). Tensile behavior of basalt textile grid reinforced Engineering Cementitious Composite. Compos. Part B Eng..

[16] Li C.H., Yin S.P., Zhao J.L. (2021). Tensile and Flexural Mechanical Properties of ECC Reinforced with Fiber Woven Mesh. J. Build. Mater..

[17] Lin J.H., Yu J.T., LIVictor C. (2016). Mechanical properties of PVA fiber reinforced engineered cementitious composites after thermal treatment. Acta Mater. Compos. Sin..

[18] Yu J., Lin J., Zhang Z., Li V.C. (2015). Experimental study on tensile properties of basalt fiber grid. Constr. Build. Mater..

[19] Jiang J.F., Dou X.X., Sui K. (2018). Experimental study on tensile properties of basalt fiber grid. Struct. Eng..

[20] Zhu J.T., Li Z.Q., Wang X.L., Li K., Liu K.W. (2021). Constitutive relationship model of engineered cementitious composites under uniaxial tension. J. Basic Sci. Eng..

